# CYP2C19 expression modulates affective functioning and hippocampal subiculum volume—a large single-center community-dwelling cohort study

**DOI:** 10.1038/s41398-022-02091-w

**Published:** 2022-08-05

**Authors:** Claire Grosu, Olga Trofimova, Mehdi Gholam-Rezaee, Marie-Pierre F. Strippoli, Ferath Kherif, Antoine Lutti, Martin Preisig, Bogdan Draganski, Chin B. Eap

**Affiliations:** 1 Unit of Pharmacogenetics and Clinical Psychopharmacology, Centre for Psychiatric Neuroscience, Department of Psychiatry, Lausanne University Hospital, University of Lausanne, Prilly, Switzerland; 2 Department of Clinical Neurosciences, Laboratory for Research in Neuroimaging LREN, Centre for Research in Neuroscience, Lausanne University Hospital, University of Lausanne, Lausanne, Switzerland; 3 Center for Psychiatric Epidemiology and Psychopathology, Department of Psychiatry, Lausanne University Hospital, University of Lausanne, Prilly, Switzerland; 4 Neurology Department, Max-Planck-Institute for Human Cognitive and Brain Sciences, Leipzig, Germany; 5 School of Pharmaceutical Sciences, University of Geneva, Geneva, Switzerland; 6 Center for Research and Innovation in Clinical Pharmaceutical Sciences, Lausanne University Hospital, University of Lausanne, Lausanne, Switzerland; 7 Institute of Pharmaceutical Sciences of Western Switzerland, University of Geneva, University of Lausanne, Lausanne, Switzerland

**Keywords:** Neuroscience, Psychology

## Abstract

Given controversial findings of reduced depressive symptom severity and increased hippocampus volume in CYP2C19 poor metabolizers, we sought to provide empirical evidence from a large-scale single-center longitudinal cohort in the community-dwelling adult population—Colaus|PsyCoLaus in Lausanne, Switzerland (*n* = 4152). We looked for *CYP2C19* genotype-related behavioral and brain anatomy patterns using a comprehensive set of psychometry, water diffusion- and relaxometry-based magnetic resonance imaging (MRI) data (BrainLaus, *n* = 1187). Our statistical models tested for differential associations between poor metabolizer and other metabolizer status with imaging-derived indices of brain volume and tissue properties that explain individuals’ current and lifetime mood characteristics. The observed association between *CYP2C19* genotype and lifetime affective status showing higher functioning scores in poor metabolizers, was mainly driven by female participants (ß = 3.9, *p* = 0.010). There was no difference in total hippocampus volume between poor metabolizer and other metabolizer, though there was higher subiculum volume in the right hippocampus of poor metabolizers (ß = 0.03, *p*_*FDR*corrected_ = 0.036). Our study supports the notion of association between mood phenotype and *CYP2C19* genotype, however, finds no evidence for concomitant hippocampus volume differences, with the exception of the right subiculum.

## Introduction

Depression is considered among the most disabling medical conditions with over 264 million people affected worldwide [1]. According to established metrics of Years Lived with Disability, it represents not only a major socio-economic burden, but it also causes great suffering of patients and their careers [1]. Any progress in understanding not only the pathophysiology, but also potential protective mechanisms are essential to both clinicians in daily practice and clinical researchers developing novel therapies.

Here, we focus on the cytochrome P450 2C19 (CYP2C19) considering previous evidence for the modulatory effect of its genetic polymorphism on brain development and individuals’ personality in later life [2, 3]. CYP2C19 is a member of the P450 superfamily, which metabolizes many drugs, xenobiotics, and endogenous compounds such as fatty acids, sex hormones such as progesterone or oestrogens [4, 5] and neurotransmitters [6, 7]. CYP2C19 is expressed during fetal development in the brain [8, 9] and after birth in the liver and gastrointestinal tract [10–12]. There is evidence from the rodent model and in humans that poor metabolizers (PM) present with milder depressive symptoms and larger hippocampus volume compared with other metabolizers (OM) [3, 8]. Previous research suggested that the CYP2C19 polymorphism is related to personality in female individuals [13], to depression traits in young males [2], and associated with basal ganglia and hippocampal volume in female individuals [14]. The assumption on brain-behavior relationships here is of a protective effect in PMs that counteracts the well-established association between depression and decreased hippocampus volume [15–19]. This interpretation was challenged by a recent study that did not find any association between *CYP2C19* genetic variation, severity of depressive or anxiety symptoms and hippocampus volume [20].

Aiming at contributing to research reproducibility, we sought to address the controversy in the literature about the differential association between CYP2C19 enzyme activity, mood phenotype and hippocampus anatomy. Here, rather than using the Hamilton Rating Scale for Depression [21] or the Beck Depression Inventory [22] that assess the current level of depression, we decided for an instrument with a lifetime perspective—the global assessment of functioning (GAF) [23], additionally to the Center for Epidemiologic Studies Depression Scale (CES-D) [24] and a diagnostic label of lifetime major depressive disorder (MDD) according to the DSM-IV [23]. Along the same lines, given major demographic and brain imaging acquisition differences between testing and validation cohorts in the literature [3], we sample data from a single-center large-scale cohort with a representative age distribution.

We used for our computational anatomy investigation relaxometry-based quantitative magnetic resonance imaging (qMRI), that holds the promise of minimizing “spurious” morphometry findings [25]. The established qMRI approach provided empirical evidence for the impact of brain tissue properties on the MRI contrast that may lead to wrong interpretation of the observed volume or cortical thickness differences in the context of brain development and aging [26, 27]. The main aim of the present study was to test and validate previous findings on the impact of CYP2C19 enzyme activity on depressive symptoms and brain anatomy. We extend further our investigation to brain tissue microstructure assessment and zoom into hippocampus subfields and associated white matter (WM) tracts. Given the controversy in the literature, our hypothesis about hippocampus anatomy differences was open, keeping in mind the low effect size of *CYP2C19* effects and potential statistical power-related differences. With a similar hypothesis, we approached the analysis of tissue properties within hippocampus subfields and associated white matter tracts.

## Methods

### Study participants

We analyze data from the CoLaus|PsyCoLaus cohort—a prospective longitudinal study designed to investigate the main effects and interactions between cardiovascular risk factors and mental disorders in the community-dwelling population. A total of 6734 individuals aged 35 to 75 years were randomly selected according to the civil register from the residents of the city of Lausanne, Switzerland, between 2003 and 2006 and underwent a physical [28, 29] and psychiatric evaluation [30]. Since the baseline assessment, there have been three completed follow-up evaluations, which took place from 2009 to 2013, 2014 to 2018, and 2018 to 2021. We included a total of 4152 individuals who had also participated in at least one psychiatric evaluation.

The computational brain anatomy analysis included all available data from the BrainLaus project (*n* = 1324) nested within the CoLaus|PsyCoLaus cohort (*n* = 4152). BrainLaus included all CoLaus|PsyCoLaus study participants who agreed to undergo an MRI and did not have any contraindications. All participants gave written informed consent, and the study was approved by the local Institutional Ethics Committee of the Canton of Vaud.

### Genotyping

DNA was extracted for studying genetic variants and biomarkers. The Colaus|PsyCoLaus participants were genotyped using the Affymetrix 500 K SNP chip (Affymetrix, Santa Clara, CA, USA) by analyzing tagging SNPs. The CYP2C19*1 allele was noted with normal enzymatic capacity, defective CYP2C19*2 allele was noted with null enzymatic capacity (poor metabolizer or PM), while the CYP2C19*17 allele was notes with increased enzymatic capacity according to the data from the previous pharmacokinetic reports [31, 32]. For more detail on enzyme activity characteristics see Table 1.

**Table 1:** CoLaus|PsyCoLaus *CYP2C19* allele description and phenotypical outcomes.

*CYP2C19* genotypes	Number (%)	CYP2C19 phenotypes	Combined CYP2C19 phenotypes
*2/*2	119 (3)	Poor metabolizers	PMs
*2/*1	849 (20)	Intermediate metabolizers	OMs
*2/*17	266 (6)	Extensive metabolizers	
*1/*1	1694 (41)	Extensive metabolizers	
*17/*1	1062 (26)	Rapid metabolizers	
*17/*17	162 (4)	Ultra rapid metabolizers	

Genetic composition of the CoLaus|PsyCoLaus.

*OMs* other metabolizers, *PMs* poor metabolizers.

### Psychometry data

We assessed individuals’ mental health using the French version of the semi-structured Diagnostic Interview for Genetic Studies (DIGS) [33, 34] conducted by trained psychologists. Global Assessment of Functioning (GAF) scores based on the DSM-IV [23] were entered by the interviewers over the participant’s lifetime, i.e., the rating took into account the severity and the duration of all psychiatric symptoms that affected the individual’s functioning over the lifespan. The GAF scores above 90 indicate superior functioning, between 90 and 70—mild impairment, and below 70—clinically significant impairment. We calculated cutoff points between dysfunctional and functional state of 70 on the GAF scale. Lifetime (trait) anxiety scores were collected using the State-Trait Anxiety Inventory (STAI) [35]. The Center for Epidemiologic Studies Depression Scale (CES-D) [24] assesses the severity of depressive symptoms during the last week. Diagnoses and MDD characteristics across the lifetime were established according to the DSM-IV [23].

### Neuroimaging data

#### qMRI data acquisition

All imaging data were acquired on the very same 3T whole‐body MRI system (Magnetom Prisma; Siemens Medical Systems, Erlangen, Germany) using a 64‐channel radiofrequency receive head coil and body coil for transmission. The qMRI protocol consisting of six to eight equidistant echo-time MT-, T1-, and PD-weighted acquisitions at spatial resolution of 1 × 1 × 1 mm [36]. According to the established biophysical model [37–39], we estimated the longitudinal relaxation rate (R1 = 1/T1) sensitive to myelin and iron content [40, 41], the effective transverse relaxation rate (R2* = 1/T2*) indicative for iron, the magnetization transfer (MT) saturation reflecting myelin content, and the effective proton density (PD*)—tissue water [42]. Before pre-processing, we corrected for the effects of B0 and B1 spatial inhomogeneities of the radiofrequency transmit field [43] and performed a quantitative quality assessment based on the level of signal degradation due to head movement using the Motion Degradation Index [44, 45].

#### qMRI data pre-processing

qMRI maps were created in the framework of voxel-based quantification (VBQ) [46, 47], for analysis of local gray and white matter volume we used voxel-based morphometry (VBM) [48]. For volume feature extraction we used SPM12s’ probabilistic tissue classification within the “unified segmentation” framework and the multi-channel option with MT and PD* maps [49], additionally to enhanced tissue priors [50]. We sampled regional volume and qMRI average values in individuals’ native space using factorization-based image labeling [51] enhanced with hippocampus subfield information [52]. Aiming to adjust all regional values for the global effect of head size, we estimated its proxy—the total intracranial volume (TIV) from the sum of gray matter, white matter, and cerebrospinal fluid (CSF) volumes [53]. Regional estimate outliers that exceeded critical threshold values of ±4 standard deviations (SD) from the region-of-interest means were excluded from the analysis. Our final analysis included 1187 participants (89.7% of the initial sample).

#### Diffusion-weighted data acquisition

The diffusion-weighted imaging (DWI) protocol consisted of a 2D echo-planar sequence at 2 mm isotropic resolution with 118 gradient directions over 3 shells with isotropic angular sampling (13 at b = 0; 15 at b = 650 s/mm^2^; 30 at b = 1000 s/mm^2^; and 60 at b = 2000 s/mm^2^) [54]. DWI data were corrected for artifacts due to eddy currents [55], subject motion and Echo-planar imaging (EPI) image distortions [56].

#### Diffusion-weighted pre-processing

For delineating WM tracts, we used the TractSeg convolutional neural network-based approach [57]. We then selected three tracts-of-interest: the fornix, the cingulum bundle, and the uncinate fasciculus. For assessment of WM microstructure, we used two established biophysical models—a diffusion tensor model and the neurite orientation dispersion and density imaging (NODDI) model [58]. Based on the tensor model, we estimated fractional anisotropy (FA) and mean diffusivity (MD) from images with b-values of 0 s/mm^2^, 650 s/mm^2^, and 1000 s/mm^2^ in MRtrix3 [59]. The NODDI model applied with the AMICO toolbox [60] estimated the orientation dispersion index (ODI), isotropic volume fraction (ISOVF), and the intracellular volume fraction (ICVF). All tensor, NODDI and qMRI metrics were sampled and averaged along the tracts-of-interest. We used the number of voxels as a proxy for tract volume. For the analyses of WM tract segmentation, we excluded 293 participants (24.6%) due to missing values and outliers’ values, which resulted in *n* = 894 participants included in the final analyses of WM tract.

### Statistical analysis

Demographic variables and psychological test scores of participants were described and compared between CYP2C19 metabolizer status using Pearson’s *χ*^2^ test of independence for categorical variables and Student’s *t* tests for continuous variables.

We hypothesized that CYP2C19 PM status would have better psychological scores. We compared lifetime GAF score in PMs with other activity scores as predicted by the genotype. We used the linear regression model to examine the association between CYP2C19 metabolizer status and the impact of mental illness symptoms on the functioning of the individual (GAF), trait anxiety (STAI), the CES-D, and logistic regression model for MDD, and we adjusted the models for the linear effects of age and sex.

Because of the involvement of CYP2C19 in the biotransformation of steroid hormones and estrogens and its association with personality trait in females [13], we investigated the main effects and interaction between sex and metabolizer status after adjusting for the linear effects of age.

We also hypothesized that CYP2C19 OM status would be reflected by hippocampal volume reduction when compared to PM individuals. We tested for association between CYP2C19 metabolizer status, hippocampus volume and hippocampal subregions (subiculum, dentate gyrus, cornu ammonis CA-1, 2, 3) using a linear regression model including age, sex, and TIV as covariates. We used identical statistical designs for the analysis of R1, R2*, MT, and PD* in GM and for MD, MT, FA, R1, R2*, ODI, ISOVF, ICVF and number of voxels in WM, including age, sex and TIV as covariates.

For statistical analyses, we used the R version 4.0.2.f software package (RStudio, Inc; Boston, Massachusetts).

We report results significant at a threshold *p* value < 0.05, and we applied False Discovery Rate (FDR) correction for multiple comparisons [61].

## Results

### CoLaus|PsyCoLaus cohort

**Fig. 1:**
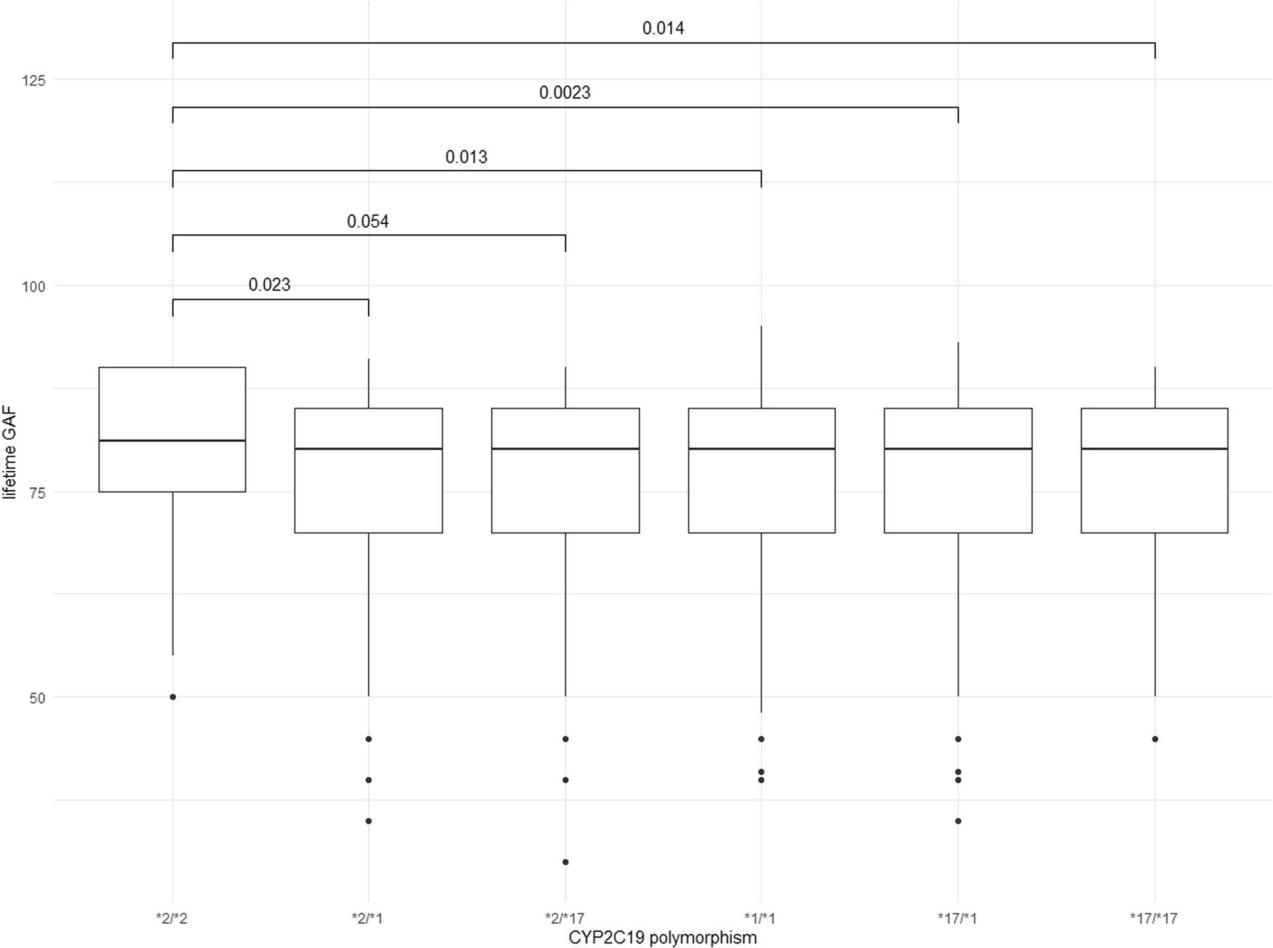
Lifetime Global Assessment of Functioning with respect of CYP2C19 polymorphism in CoLaus|PsyCoLaus (*n* = 4152). Boxplots showing that the increase in lifetime global assessment of functioning score is limited to poor metabolizers (*2/*2). Boxplots showing the median GAF and standard deviation for each *CYP2C19* allelic form. GAF global assessment of functioning.

There were no statistical differences in age and sex between the OM and the PM participants (Table 2). *T* tests revealed that PMs showed higher lifetime GAF score compared to OMs (PM: 80 ± 9.4; OM: 77 ± 12; *t*_(4152)_ = −3.6; *p* < 0.001), indicating better global functioning (Table 2). This difference in lifetime GAF score was still significant when PMs were compared with other activity scores as predicted by the genotype (Fig. 1) showing that the effect is limited to PM status. There were no other psychometry differences.

**Table 2:** Effects of the *CYP2C19* genotype and sex on psychological scores in CoLaus|PsyCoLaus (*n* = 4152).

	OM	PM	Interaction		Age and sex adjusted associations		
			genotype × sex				
	(*n* = 4033)	(*n* = 119)	ß/OR (95% CI)	*p*	ß/OR (95% CI)	*p*	*p FDR*
Sex (male), no. (%)	1848 (46)	61 (51)	–	–	–	–	
Age, mean (SD)	55 (11)	55 (11)	–	–	–	–	
GAF lifetime							
All, mean (SD)	77 (12)	80 (9.4)	ß = 1.7 (0.19, 5.8)	0.045*	ß = 3.0 (1.1, 5.1)	0.003	0.018*
Male, mean (SD)	80 (11)	81 (9.4)			ß = 2.2 (−0.6, 5.0)	0.12	
Female, mean (SD)	76 (12)	80 (9.4)			ß = 3.9 (0.87, 7.0)	0.010*	
GAF current							
All, mean (SD)	80 (14)	81 (13)	ß = 0.90 (−4.2, 6.0)	0.73	ß = 1.5 (−1.0, 4.1)	0.24	ns
Male, mean (SD)	81 (13)	82 (12)					
Female, mean (SD)	78 (15)	80 (14)					
GAF worst							
All, mean (SD)	57 (19)	59 (20)	ß = 0.89 (−6.0, 7.8)	0.8	ß = 2.1 (−1.3, 5.6)	0.22	ns
Male, mean (SD)	60 (20)	62 (21)					
Female, mean (SD)	54 (19)	57 (18)					
STAI trait							
All, mean (SD)	36 (11)	35 (9.3)	ß = 1.8 (−3.0, 6.7)	0.46	ß = −1.9 (−5.3, 1.6)	0.29	ns
Male, mean (SD)	34 (10)	32 (7.8)					
Female, mean (SD)	37 (11)	37 (10)					
CES-D							
All, mean (SD)	11 (8.8)	9.8 (7.0)	ß = 2.0 (−1.3, 5.4)	0.23	ß = −1.9 (−4.3, 0.51)	0.12	ns
Male, mean (SD)	9.4 (8.1)	7.6 (5.3)					
Female, mean (SD)	12 (9.1)	12 (7.8)					
MDD (yes)							
All, no. (%)	1614 (40)	43 (36)	OR = 0.57 (−2.1, 2.8)	0.15	OR = 1.2 (0.67, 2.0)	0.56	ns
Male, no. (%)	543 (29)	20 (33)					
Female, no. (%)	1071 (49)	23 (40)					

Values in the second and third columns are reported as the number of patients (no.), as percentage (%) or as the mean with standard deviation. The fourth and fifth columns reported the beta coefficients and odd ratios with *p* values for the interaction tests between genotype and sex. In columns six and seven, beta coefficients and *p* values are reported from linear regression models; odd ratios and *p* values are reported from logistic regression models; scores were individually regressed against poor metabolizer status, including age and sex as covariates.

*CES-D* center for epidemiologic studies depression scale, *FDR* false discovery rate, *GAF* global assessment of functioning, *MDD* major depressive disorder, *ns* not significant, *OM* other metabolizer, *PM* poor metabolizer, *SD* standard deviation, *STAI* state and trait self-reported anxiety scores.

*P < 0.05.

In the linear model analysis, we observed an effect of CYPC219 metabolizer status on lifetime GAF score (ß = 3.0, 95% CI: 1.0–5.1, *p*_FDRcorrected_ = 0.018, Table 2).

This observation remained significant after excluding the MRI participants from the analysis (*n* = 2965; OMs *n* = 2879, PMs *n* = 86, mean lifetime GAF score in OM—77 ± 12; in PM—80 ± 10; t_(2965)_ = −2.7; *p* = 0.009, data not shown). In addition, we found an association with the linear model between lifetime GAF and CYP2C19 metabolizer status when corrected by age and sex (ß = 2.9, 95% CI: 0.43–5.4, *p* = 0.022, data not shown).

To exclude the possibility that another factor is affecting the lifetime GAF scores, we adjusted the analyses for mental diseases other than depression or anxiety disorders (e.g., substance use schizoaffective, psychotic, and bipolar disorders): the effect of CYP2C19 metabolizer status on lifetime GAF score remained significant (ß = 2.9, 95% CI: 0.84–5.0, *p* = 0.006, data not shown).

We found an interaction effect between CYP2C19 metabolizer status and sex (ß = 1.7, 95% CI: 0.19–5.8, *p* = 0.045, Table 2) on the lifetime GAF score. A post hoc analysis showed that females were the main drivers of the association between GAF scores and CYP2C19 metabolizer status (ß = 3.9, 95% CI: 0.87–7.0, *p* = 0.010, Table 2). There were no significant results in the identical models testing for the interaction between CYPC219 metabolizer status, sex and CES-D, current and worse GAF, trait-STAI- scores and MDD diagnosis.

### BrainLaus sample

In the BrainLaus sample, with PMs (*n* = 33) and OMs (*n* = 1154), *t* tests showed higher lifetime GAF scores in PMs (PM: 82 ± 8; OM: 78 ± 11; *t*_(1187)_ = −2.8; *p* = 0.008, data not shown) when compared to OMs.

The linear regression showed a trend with borderline significance between lifetime GAF and CYP2C19 metabolizer status association (ß = 3.5, 95% CI: −0.2–7.1; *p* = 0.063, Supplementary Table 1).

There was also an association between CYP2C19 metabolizer status in BrainLaus and the lifetime GAF in female participants (ß = 7.1, 95% CI: 1.4–13; *p* = 0.018, Supplementary Table 1) but not in male individuals (ß = 0.5, 95% CI: −4.3–5.2; *p* = 0.84, Supplementary Table 1).

### Hippocampus and hippocampal subregions

There was no significant effect of CYP2C19 metabolizer status on hippocampal volumes, MT, R1, R2*, and PD* regional averages (Supplementary Table 2). The analysis of hippocampal subregions showed higher right subiculum volume in PM participants (ß = 0.03, 95% CI: 0.01–0.05, *p*_*FDRcorrected*_ = 0.036, Table 3). This result was not paralleled by differences in MT, R1, R2*, or PD* regional averages.

**Table 3:** Univariate associations between hippocampal volumes and CYP2C19 status after adjusting for age, sex, and total intracranial volume in BrainLaus (*n* = 1187; 33 poor metabolizers, 1154 other metabolizers).

	PM status			
	ß	95% CI	*p*	*p FDR*
Left subiculum	0.02	−0.01, 0.05	0.12	0.36
Right subiculum	0.03	0.01, 0.05	0.006*	0.036*
Left dentate gyrus	0.0002	−0.01, 0.01	0.91	0.91
Right dentate gyrus	0.001	−0.01, 0.01	0.59	0.86
Left CA123	0.01	−0.05, 0.06	0.72	0.86
Right CA123	0.04	−0.03, 0.10	0.26	0.52

Beta coefficients and *p* values are reported from linear regression models where behavioral scores were individually regressed against poor metabolizer status, including age sex and total intracranial volume as covariates.

*ß* beta coefficients, *CA123* cornu ammonis 123, *CI* confidence interval, *FDR* false discovery rate, *PM* poor metabolizer.

*p < 0.05.

### Hippocampus-centered white matter tracts

In the tracts-of-interest-cingulate, uncinate fasciculus and fornix, we report a significant effect of CYP2C19 metabolizer status on the orientation dispersion index in the left cingulum bundle (ß = −0.50, 95% CI: −0.88 to −0.12, *p*_*uncorrected*_ = 0.010, Supplementary Table 3) and in the left uncinate fasciculus (ß = −0.49, 95% CI: −0.87 to −0.11, *p*_*uncorrected*_ = 0.012, Supplementary Table 4). However, we lose these associations after applying the FDR correction for multiple comparisons.

## Discussion

In our study investigating the impact of *CYP2C19* genotype on mood phenotype and brain anatomy, we validated previous findings showing an impact of CYP2C19 metabolizer status on global measures of mood. The absence of *CYP2C19* determined enzymatic activity was related to superior mental health assessed with the lifetime GAF score, which was mainly driven by female participants. The effects of CYP2C19 metabolizer status on brain anatomy were confined to higher right subiculum volume in PMs. Given the fact that *CYP2C19* is expressed in the brain during the prenatal period, we interpret the obtained results as a confirmation for the long-lasting impact of this genotype on brain anatomy and affective behavior.

We complement previous reports to show an effect of CYP2C19 enzymatic activity on lifetime assessment of mood and global functioning beyond the impact on current affective state. This is a novel finding that sheds light on the published controversial results in humans [14, 20]. We show a specific CYP2C19 enzymatic activity effect confined to the right hippocampal subiculum. The widespread subiculum projections reach cortical areas related to stress response and depression [62] to form regulatory hubs for hippocampus-cortical communication [63]. The lack of differences in our analysis when averaging the volume across the whole hippocampus is at odds with the findings in a similarly well-powered cohort focusing on young participants (mean age 37.3 years) [3], and in another cohort consisting of female participants (*n* = 342; mean age 24.1 years) [14], but partially confirmatory to another less well-powered report [20]. The controversial result may stem from a broad range of factors: i. ascertainment method—sampling in the general population vs. adverts, additionally to mono- vs. multicentric; ii. statistical design—unaccounted linear and non-linear effects of age, socio-economic status etc. iii. neuroimaging protocol—relaxometry-based vs. T1-weighted MRI susceptible to “spurious” morphometric findings [25], to name but a few.

Our findings of reduced ODI—index of neurite dispersion, in the left cingulum bundle and left uncinate fasciculus are indicative for higher tract coherence in PMs. However, due to the loss of significant effect after multiple testing correction, replication in another large study cohort is required to validate these results. This was contrasted by the lack of similar differences in the fornix that we explain with its small volume that does not show interindividual variability in tract dispersion [63].

The finding of interaction effects of CYP2C19 enzymatic activity and sex on lifetime GAF scores is novel. The interpretation of associations between metabolizer status and affective behavior within a cross-sectional analysis framework is challenging. These could be related to the established higher incidence of major depression in females (5.5%) than males (3.2%) [64]. Among Colaus|PsyCoLaus participants with depressive disorder, females represent 57% [65]. On the other hand, a lower CYP2C19’s metabolic activity could be associated with a higher oestrogen level that would explain the observed differential effects across sexes [4, 5].

We acknowledge several limitations of our study. First, whereas past studies have shown a causal link between behavior and brain volumes in *CYP2C19* knock-in mice [8, 9], the present study tests only associations without any pretention for a link to causality. Second, given the nature of our study aiming at investigating aging effects, younger age groups remain underrepresented [28]. This becomes clear when comparing the mean age of our cohort (mean ± SD: 53.4 ± 9.4 years) with the aforementioned previous studies (37.3 ± 11.6 years [3] and 23.6 ± 5.3 years [20]). As depressive disorders are typically more prevalent in younger than in older age groups [66], the underrepresentation of younger individuals (with depression) is likely to impact our results.

In summary, we report associations between CYP2C19 metabolizer status and measures of lifetime mood and global functioning, with PMs having higher scores than OMs that were mainly driven by female participants. The brain anatomy correlates of this difference were higher right hippocampal subiculum volume. We interpret our findings as behavioral and brain anatomy fingerprints of the presumed protective effect of absent CYP2C19 activity on mood in humans.

## Supplementary information

SUPPLEMENTARY FILES
