# Supplementary material for: CYP2C19 expression modulates affective functioning and hippocampal subiculum volume—a large single-center community-dwelling cohort study
Source: Transl Psychiatry. 2022 Aug 5;12:316. doi: 10.1038/s41398-022-02091-w (PMC9356029; doi:10.1038/s41398-022-02091-w)
Supplement: Supplementary file 1 — SUPPLEMENTARY FILES [file 41398_2022_2091_MOESM1_ESM.docx]

**CYP2C19 expression modulates affective functioning and hippocampal subiculum volume – a large single-center community-dwelling cohort study**

**SUPPLEMENTARY FILES**

Claire Grosu^1^; Olga Trofimova^2^; Mehdi Gholam-Rezaee^3^; Marie-Pierre F. Strippoli^3^; Ferath Kherif^2^; Antoine Lutti^2^; Martin Preisig^3^; Bogdan Draganski^2,4^*; Chin B. Eap^1,5,6,7^*.

*^1^Unit of Pharmacogenetics and Clinical Psychopharmacology, Centre for Psychiatric Neuroscience, Department of Psychiatry, Lausanne University Hospital, University of Lausanne, Prilly, Switzerland*

*^2^Laboratory for Research in Neuroimaging LREN, Centre for Research in Neuroscience - Department of Clinical Neurosciences, Lausanne University Hospital and University of Lausanne, Switzerland*

*^3^Center for Psychiatric Epidemiology and Psychopathology, Department of Psychiatry, Lausanne University Hospital, University of Lausanne, Prilly, Switzerland*

*^4^Neurology Department, Max-Planck-Institute for Human Cognitive and Brain Sciences, Leipzig, Germany*

*^5^School of Pharmaceutical Sciences, University of Geneva, Geneva, Switzerland.*

*^6^Center for Research and Innovation in Clinical Pharmaceutical Sciences, Lausanne University Hospital and University of Lausanne, Switzerland*

*^7^Institute of Pharmaceutical Sciences of Western Switzerland, University of Geneva, University of Lausanne*

** equal contribution*

Running title: CYP2C19, brain anatomy and behavior

Keywords: Behavior, CYP2C19, Global Assessment of Functioning, Hippocampus, Metabolizers, Subiculum.

Corresponding authors:

Chin B. Eap Bogdan Draganski

Centre de neurosciences psychiatriques LREN

Hôpital de Cery, Route de Cery 11 B Chemin de Mont-Paisible 16

CH-1008 Prilly-Lausanne CH-1010 Lausanne

Email: [Chin.Eap@chuv.ch](mailto:Chin.Eap@chuv.ch) Email: [Bogdan.Draganski@chuv.ch](mailto:Bogdan.Draganski@chuv.ch)

Phone: +41 21 314 26 04 Phone: +41 21 314 96 38

**Supplementary Table 1.** Univariate associations between lifetime global assessment of functioning scores and CYP2C19 status in BrainLaus (n=1187; 33 poor metabolizers, 1154 other metabolizers) in males (n=592; 18 poor metabolizers, 574 other metabolizers) and females (n=595; 15 poor metabolizers, 580 other metabolizers).

| **Psychological scores** | **PM Genotype** | | | | **Sex** | | | | **Age** | | |  |
| --- | --- | --- | --- | --- | --- | --- | --- | --- | --- | --- | --- | --- |
|  | **ß** | **95% CI** | ***p*** | **ß** | | **95% CI** | ***p*** | **ß** | | **95% CI** | ***p*** |  |
| **GAF Lifetime (all ; n=1187)** | 3.5 | -0.2, 7.1 | 0.063 | -2.8 | | -4.0, -1.6 | <0.001* | 0.1 | | 0.06, 0.18 | <0.001* |  |
| **GAF Lifetime (male ; n=592)** | 0.5 | -4.3, 5.2 | 0.84 | - | | - | - | 0.1 | | 0.01, 0.17 | 0.03* |  |
| **GAF Lifetime (female ; n=595)** | 7.1 | 1.4, 13 | 0.018* | - | | - | - | 0.1 | | 0.06, 0.23 | 0.001* |  |

Beta coefficients and *p*-values are reported from linear regression model where lifetime global assessment of functioning score was regressed against poor metabolizer status, including age and sex or only age as covariates.

ß: beta coefficient; CI: confidence interval; GAF: global assessment of functioning; PM: poor metabolizer. **p*<0.05.

**Supplementary Table 2.** Univariate associations between right and left hippocampus volumes and tissue properties and CYP2C19 status in BrainLaus (n=1187; 33 poor metabolizers, 1154 other metabolizers).

|  | **PM Status (Right Hippocampus)** | | | **PM Status (Left Hippocampus)** | | |
| --- | --- | --- | --- | --- | --- | --- |
|  | **ß** | **95% CI** | ***p*** | **ß** | **95% CI** | ***p*** |
| **Hippocampus GM Volume (mm3)** | 0.06 | -0.02, 0.15 | 0.12 | 0.03 | -0.05, 0.11 | 0.4 |
| **Hippocampus PD*** | -0.19 | -0.60, 0.22 | 0.4 | -0.10 | -0.53, 0.34 | 0.7 |
| **Hippocampus MT** | -0.01 | -0.02, 0.01 | 0.4 | 0.005 | -0.01, 0.02 | 0.6 |
| **Hippocampus R1** | -0.07 | -11, 11 | 0.9 | -2.9 | -13, 7.6 | 0.6 |
| **Hippocampus R2*** | 0.0001 | -0.004, 0.006 | 0.7 | 0.00001 | -0.0004, 0.0004 | 0.9 |

Beta coefficients and p-values are reported from linear regression models where grey matter volumes were individually regressed against poor metabolizer status, including age, sex and total intracranial volume as covariates.

ß: beta coefficient; CI: confidence interval; GM: grey matter; MT: magnetization transfer; PD*: proton density; PM: poor metabolizer; R1: longitudinal relaxation rate; R2*: effective transverse relaxation rate.

**Supplementary Table 3.** Univariate associations between right and left cingulum bundles tissue properties and CYP2C19 status in BrainLaus (n=894; 27 poor metabolizers, 867 other metabolizers).

|  |  | **PM status (Right Cingulum Bundle)** | | | **PM status (Left Cingulum Bundle)** | | |
| --- | --- | --- | --- | --- | --- | --- | --- |
|  |  | **ß** | **95% CI** | ***p_uncorrected_*** | **ß** | **95% CI** | ***p_uncorrected_*** |
| **Number of voxels** | | -120 | -415, 174 | 0.42 | -130 | -336, 76 | 0.21 |
| **Mean MD** | | 0.26 | -0.12, 0.65 | 0.17 | 0.16 | -0.17, 0.49 | 0.34 |
| **Mean MT** | | -0.27 | -0.65, 0.11 | 0.17 | -0.04 | -0.37, 0.29 | 0.80 |
| **Mean FA** | | -0.23 | -0.62, 0.15 | 0.23 | 0.18 | -0.17, 0.53 | 0.31 |
| **Mean R1** | | -11 | -28, 5.2 | 0.18 | -9.6 | -29, 9.5 | 0.32 |
| **Mean R2*** | | -0.23 | -0.61, 0.14 | 0.22 | -0.15 | -0.50, 0.20 | 0.41 |
| **Mean ODI** | | -0.08 | -0.46, 0.31 | 0.69 | -0.50 | -0.88, -0.12 | 0.010** |
| **Mean ICVF** | | -0.24 | -0.62, 0.15 | 0.23 | -0.09 | -0.45, 0.26 | 0.60 |
| **Mean ISOVF** | | 0.08 | -0.31, 0.46 | 0.69 | 0.12 | -0.23, 0.47 | 0.50 |

Beta coefficients and uncorrected p-values are reported from linear regression models where white matter properties were individually regressed against poor metabolizer status, including age, sex, and total intracranial volume as covariates.

ß: beta coefficient; CI: confidence interval; FA: fractional anisotropy; ICVF: intracellular volume fraction; ISOVF: isotropic volume fraction; MD: mean diffusivity; MT: magnetization transfer; ODI: orientation dispersion index; PM: poor metabolizer; R1: longitudinal relaxation rate; R2*: effective transverse relaxation rate. ***p*<0.05

**Supplementary Table 4.** Univariate associations between right and left uncinate fasciculus tissue properties and CYP2C19 in BrainLaus (n=894; 27 poor metabolizers, 867 other metabolizers).

|  |  |  | **PM status (Right Uncinate Fasciculus)** | | | **PM status (Left Uncinate Fasciculus)** | | |
| --- | --- | --- | --- | --- | --- | --- | --- | --- |
|  |  |  | **ß** | **95% CI** | ***p_uncorrected_*** | **ß** | **95% CI** | ***p_uncorrected_*** |
| **Number of voxels** | | | -46 | -162,71 | 0.44 | 9.9 | -97, 117 | 0.85 |
| **Mean MD** | | | 0.29 | -0.09, 0.68 | 0.13 | 0.01 | -0.38, 0.39 | 0.99 |
| **Mean MT** | | | -0.10 | -0.48, 0.28 | 0.61 | 0.27 | -0.11, 0.65 | 0.16 |
| **Mean FA** | | | -0.12 | -0.51, 0.26 | 0.52 | 0.35 | -0.04, 0.73 | 0.076 |
| **Mean R1** | | | -3.7 | -20, 13 | 0.66 | 13 | -4.6, 30 | 0.2 |
| **Mean R2*** | | | -0.12 | -0.50, 0.27 | 0.55 | 0.32 | -0.06, 0.71 | 0.10 |
| **Mean ODI** | | | -0.31 | -0.69, 0.07 | 0.11 | -0.49 | -0.87, -0.11 | 0.012** |
| **Mean ICVF** | | | -0.28 | -0.66, 0.11 | 0.16 | 0.11 | -0.27, 0.50 | 0.56 |
| **Mean ISOVF** | | | 0.13 | -0.26, 0.51 | 0.52 | 0.11 | -0.27, 0.50 | 0.56 |

Beta coefficients and uncorrected p-values are reported from linear regression models where white matter properties were individually regressed against poor metabolizer status, including age, sex and total intracranial volume as covariates.

ß: beta coefficient; CI: confidence interval; FA: fractional anisotropy; ICVF: intracellular volume fraction; ISOVF: isotropic volume fraction; MD: mean diffusivity; MT: magnetization transfer; ODI: orientation dispersion index; PM: poor metabolizer; R1: longitudinal relaxation rate; R2*: effective transverse relaxation rate. ***p*<0.05
